# Peripheral primitive neuroectodermal tumor of the parotid gland in a child: A case report

**DOI:** 10.3892/ol.2014.2156

**Published:** 2014-05-19

**Authors:** XING WANG, JIAN MENG

**Affiliations:** 1 Department of Stomatology, Xuzhou Clinical School of Xuzhou Medical College, Xuzhou, Jiangsu 221000, P.R. China; 2 Department of Dentistry, Central Hospital of Xuzhou City, Xuzhou, Jiangsu 221009, P.R. China

**Keywords:** peripheral primitive neuroectodermal tumor, parotid gland, child, mumps, misdiagnose

## Abstract

Primitive neuroectodermal tumor (PNET) is a term used to describe a group of highly malignant neoplasms of soft-tissue origin, with varying degrees of divergent differentiation. The occurrence of peripheral PNET in the head and neck region has been reported infrequently in the medical literature. This disease generally occurs in adolescents and young adults, and rarely occurs in children <3 years old. The current study presents an extremely rare case of pPNET of the parotid gland in a 2-year-old male, which had been previously misdiagnosed and treated as a mumps. The lesion showed the characteristic histological features of pleomorphic cellular infiltrate with hyperchromatic small cells scattered in the fibrovascular stroma, interposed by fibrous septa and Homer-Wright rosettes. Positive immunohistochemical staining for CD99 and vimentin was detected. The patient was treated with chemotherapy and radiotherapy following surgical removal, and has been under close observation since the treatment (approximately seven months), with no signs of recurrence. The clinical history and radiological and histopathological findings are presented, together with the immunoreactivity of this tumor.

## Introduction

Primitive neuroectodermal tumors (PNETs) are rare small round cell neoplasms, first described in 1918 by Stout (1) as a malignant tumor arising from major nerve (2). All ages are affected, but the majority of patients present in their first or second decades of life, and the tumors are rarely observed in children <3 years old.

Although peripheral PNET (pPNET; PNET derived from tissues outside the central and autonomic nervous systems) frequently occurs in the thoracopulmonary region (Askin’s tumors), urogenital tract and testis (3–4). pPNET has also been found in the head and neck region (6). pPNET comprises 1% of all sarcomas, and is highly malignant (7). Clinically, the condition presents as a rapidly enlarging, painful mass, with a high probability of micrometastasis (6).

The symptoms of pPNET in the head and neck are non-specific. An extensive review of the literature shows only a few cases of pPNET affecting the parotid gland (8). To the best of our knowledge, the present study reports the first case of pPNET of the parotid gland affecting a child <3 years old, which had been previously misdiagnosed and treated as mumps. Written informed consent was provided by the patient’s family.

## Case report

A 2-year-old male, with no noteworthy medical history, was brought to the Department of Oral Surgery of Xuzhou Central Hospital (Xuzhou, China) with a 1-month history of a painful, progressively enlarging swelling in the left parotid gland accompanied by cervical lymphadenopathy, which had previously been treated as mumps by a county hospital. Prior to this referral, the patient was treated with antibiotics for 1-week. The patient had no noteworthy medical family history or past history. Upon physical examination, the patient was afebrile, but anorexic. Upon clinical examination, a firm non-tender fixed mass (5×5 cm) was found on the left parotid gland extending to the parapharyngeal space, without facial paralysis.

**Figure 1:**
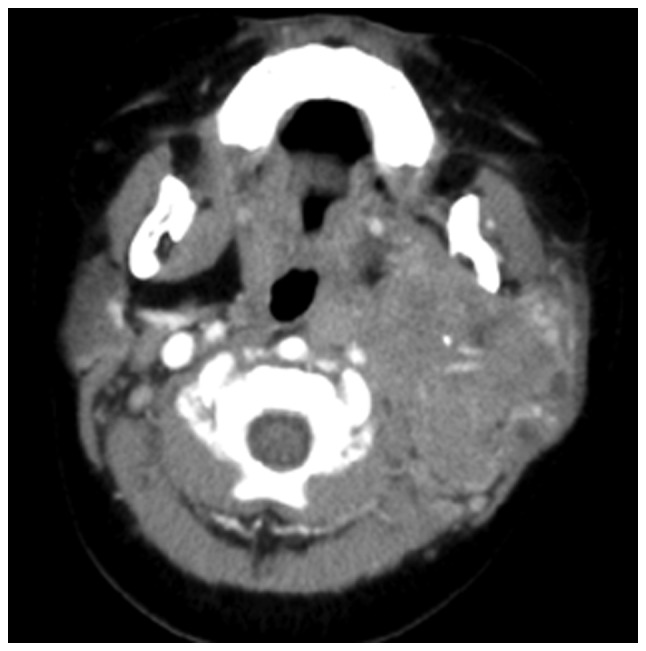
Axial computed tomography (CT) scan showing a large heterogenous mass in the left parotid region crossing the carotid arteries, without the underlying bone.

A computed tomography (CT) scan of the head-neck was indicative of a soft-tissue mass of heterogenous density in the left side of the parotid gland completely surrounding the carotid arteries (Fig. 1). The mass was ~5.6×4.8×5.3 cm in size and invaded the left parapharyngeal space and lateral pterygoid plate without bone erosion. These observations were confirmatory for a diagnosis of a PNET. A chest radiograph and abdominal ultrasound were performed to screen for metastasis, and revealed normal results.

**Figure 2:**
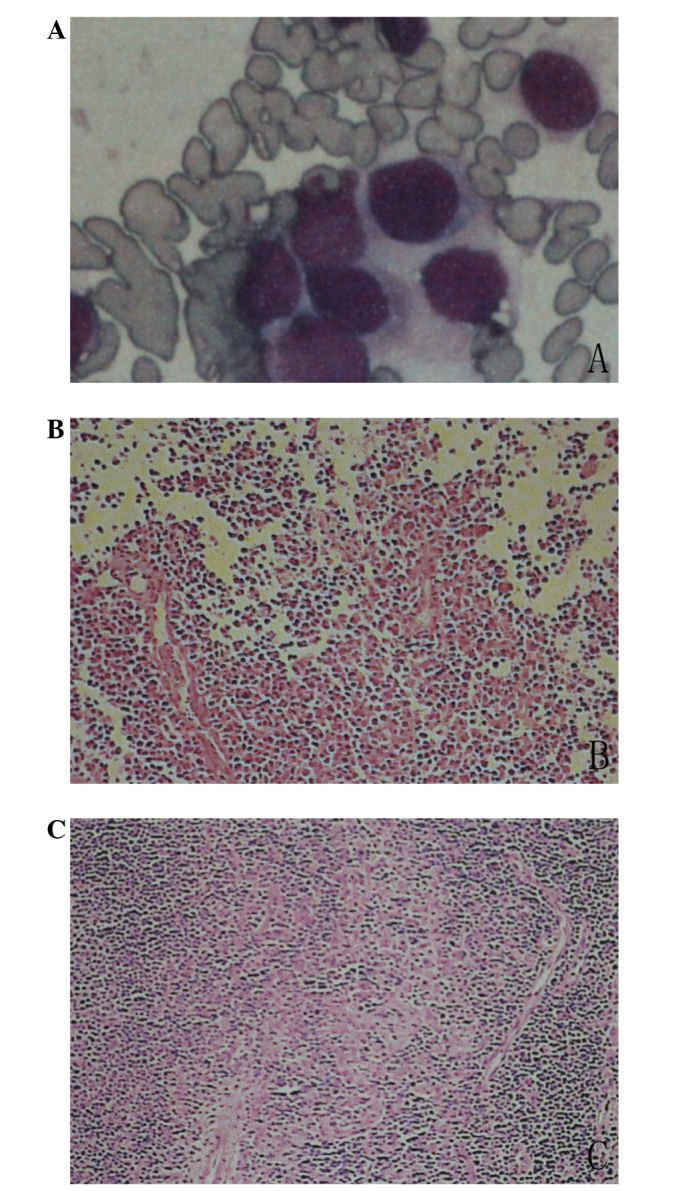
(A) A low-power view of the biosy specimen showing a pleomorphic cellular infiltrate with hyperchromatic small cells (hematoxylin and eosin staining; original magnification, ×40). (B) Sheets of tumor cells exhibiting numerous Homer-Wright-type rosettes (hematoxylin and eosin staining; original magnification, ×10). (C) Tumor cell separated by fibrous septa (hematoxylin and eosin staining; original magnification, ×10).

**Figure 3:**
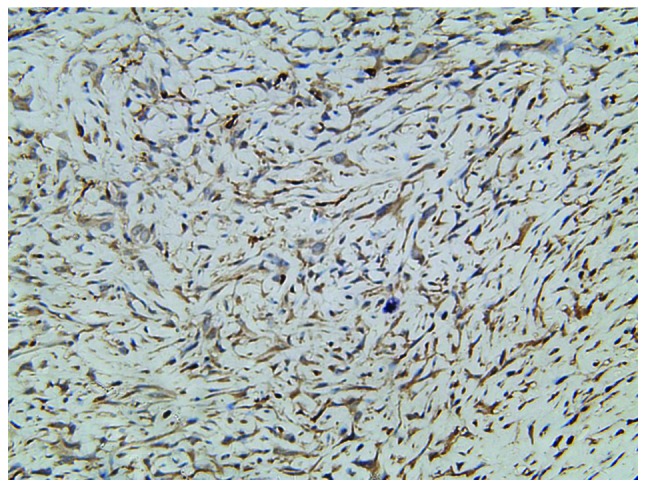
Immunohistochemical staining for cluster of differentiation (CD)99 revealing strong, diffuse positivity of the tumor cells (hematoxylin and eosin staining; original magnification, ×20).

Owing to the requirement for obtaining a histopathological diagnosis, the patient underwent a needle aspiration biopsy and excisional biopsy for analysis. Microscopically, the biopsy specimen exhibited a pleomorphic cellular infiltrate with hyperchromatic small cells scattered in a fibrovascular stroma interposed by fibrous septa. Homer-Wright rosettes consisting of a number of hyperchromatic cells were also present (Fig. 2). Definitive management in the form of debulking surgery and parotidectomy was performed in December 2012. Following the surgery, an immunohistochemical analysis was performed on the excised material for a definite diagnosis. The sample was positive for cluster of differentiation (CD)99 (Fig. 3), synaptophysin (Syn), vimentin, chromogranin A (CgA) and cytokeratin (CK). Ki-67 (mib-1) immunochemical staining showed 50% of positive cells. Other immunomarkers, including desmin, epithelial membrane antigen and CD38, were all negative. On the basis of these findings, the lesion was confirmed to be a pPNET of the parotid gland.

Subsequent to the generation of a definite diagnosis, the patient was started on radiotherapy (20 Gy) treatment and multiagent chemotherapy, including cyclophosphamide, adriamycin and vincristine (CAV). The mass was grossly reduced in size, but did not completely disappear. Chest CT and abdominal ultra sonography were performed every 3 months. However, following the third chemotherapeutic cycle, the patient experienced severe vomiting and diarrhea, and the parents refused to continue the treatment. The patient is currently being monitored by follow-up examinations at 7 months post-surgery.

## Discussion

PNET belongs to the Ewing family of tumors. In 1918, Stout (1) first described the tumor composed of small-round cells in the ulnar nerve (2). PNET is frequently observed in adolescents, and can be found anywhere within the body, particularly in the trunk and extremities. Although pPNET is rarely noted in the head and neck region, when found, it is often in the jaw, followed by the mandible and maxilla. pPNET of the parotid gland is exceedingly unusual. In a review of the English literature, it was found that pPNET of the parotid gland had only been reported in adults, aged between 15 and 60 years old (7). Therefore, to the best of our knowledge, the present case of pPNET of the parotid gland is the first study in a child.

Clinically, the majority of complaints by patients at the time of presentation include rapid growth, swelling of the affected area and pain, all complaints associated with the soft mass effects of the tumor (6–8). However, pre-operative diagnosis is difficult, as clinical presentation may vary greatly between the different sites of involvement. In children, when parotid gland swelling occurs with other systemic signs, such as fever, anorexia, fatigue and respiratory symptoms, the condition may be misdiagnosed and treated as mumps (9). The patient in the current case presented with a sudden onset of parotid swelling accompanied by anorexia and pain. The patient had previously been misdiagnosed by a county hospital and only treated with antibiotics. According to the study by Brazão-Silva *et al* (10), the complementary examinations avoid larger areas in the diagnosis, which may affect the early diagnosis and lead to the risk of more aggressive approaches. We believe that improved diagnostic techniques and therapeutic strategies for patients with pPNET could increase the disease-free survival time.

Imaging studies are essential for the diagnosis and surgical treatment planning, however, the radiological features of the tumors are non-specific and frequently cannot be differentiated from those of other types of bone and soft-tissue tumors (11). Although pPNET most often resemble large, non-calcified, soft-tissue masses, with cystic or necrotic areas, this aspect is not a pathognomonic feature, as other lesions can have the same image pattern. Magnetic resonance imaging also reveals a non-specific isointensity on T1/T2-weighted images (11–13). Ultimately, a diagnosis is made by histopathological and immunohistochemical examination of the tumor.

Histologically, pPNET consists of a pleomorphic cellular infiltrate with hyperchromatic small cells, which are often arranged in lobules separated by fibrous septa (14). In addition, short dendritic processes lie between the cells in a pPNET, and these are characteristically absent in Ewing’s sarcoma (10). Homer-Wright-type rosettes with tumor cells are absent in PNET, which is seen in other round cell tumors. However, due to their similarity with numerous tumors belonging to the category of ‘small, round, blue cell tumors’, including rhabdomyosarcoma, lymphoma and small cell carcinoma, as well as other members of the PNET family of tumors, particularly neuroblastoma and Ewing’s sarcoma, which exhibit a similar histological appearance, the diagnosis of pPNETs can be problematic on a morphological basis. The use of immunohistochemistry plays a pivotal role in the diagnosis of this tumor. Normally, the tumor cells are positive for MIC-2 (CD99) and vimentin, and negative for desmin and CK, but are not specific markers for pPNET. Schmidt *et al* (15) proposed the following criteria for the pathological diagnosis of PNET: a) Tumor cells appear round under a light microscope and Homer-Wright-type rosettes are present; b) immunohistochemistry reveals that the tumor is positive for 013 (i.e., HBA71, P30/32 and MIC-2), and for two or more of the NSE, Syn and CGA proteins; and c) ultrastructural identification of neural elements, especially neurosecretory granules (14,16).

Due to the rare occurrence of pPNET, optimal therapy is challenging, particularly if the tumors occur in the head and neck. Currently, the uniformly accepted strategy is surgery combined with adjuvant radiotherapy and multiagent chemotherapy (17,18). However, structural complexity of the head and neck region leads to difficulty in complete removal. We believe that close cooperation between surgeons, oncologists and radiotherapists can improve progression-free survival times. The patient of the present study underwent maximal tumor reduction surgery, CAV and low-dose pre-operative radiation therapy for gross residual disease or microscopic residual disease. The patient has been under close observation since the treatment (~7 months), and there have been no signs of recurrence.

In conclusion, the differential diagnosis of pPNET in a child is extremely important. In the present case, it was noted that combination therapy can be also be an effective method for young children with pPNET, while at the same time, attention must be paid to adverse reactions.
